# Detecting Familial hypercholesterolemia in children and adolescents: potential and challenges

**DOI:** 10.1186/s13052-022-01257-y

**Published:** 2022-07-15

**Authors:** Giuseppe Banderali, Maria Elena Capra, Giacomo Biasucci, Rita Stracquadaino, Claudia Viggiano, Cristina Pederiva

**Affiliations:** 1 Department of General Paediatrics, Clinical Dyslipidemia Service for the Study and Prevention of Atherosclerosis in Children, ASST-Santi Paolo E Carlo, University of Milan, Milan, Italy; 2 Centre for Paediatric Dyslipidaemias, Paediatrics and Neonatology Unit, Guglielmo da Saliceto Hospital, 29121 Piacenza, Italy; 3 Department of Translational Medical and Surgical Sciences, University of Parma, 43126 Parma, Italy

**Keywords:** Familial hypercholesterolemia, Children, Cardiovascular disease, Screening

## Abstract

**Background:**

It is now well established that atherosclerosis begins in childhood and evolves through adolescence and young adulthood, ultimately resulting in myocardial infarction and stroke in adults.

**Main test:**

Childhood is a critical phase during which atherosclerosis may begin to develop; in the presence of familial hypercholesterolemia, lifelong elevation of Low Density Lipoprotein cholesterol levels greatly accelerates atherosclerosis. These concepts, which have been largely developed from epidemiologic evidence, have not always been simple to implement in the paediatric clinical practice.

The purpose of this article is to briefly review but also to highlight the rationale, the motivation and the methods in the process of identifying children and adolescents with familial hypercholesterolemia, an often hidden but very important genetic disease.

## Background

Familial hypercholesterolemia (FH) is a common disorder of lipid metabolism, and the most common inherited metabolic disease. It is characterized by elevated serum levels of total cholesterol (TC) and low-density lipoprotein cholesterol (LDL-C), leading to premature coronary heart disease (CHD) if left untreated. Due to a genetic defect in the low-density lipoprotein (LDL)-receptor (LDLR) pathway, affected patients cannot clear LDL particles from the circulation; as a consequence, life-long accumulation of low-density lipoprotein cholesterol (LDL-C) in plasma and early atherosclerosis may occur. The cumulative cholesterol burden is much greater in homozygous FH (HoFH), and such patients develop severe life-threatening CHD and other vascular complications in late childhood and adolescence if not recognized and treated [1]. The underlying defect is, in almost 90% of the genetically diagnosed FH patients, a mutated LDLR, which results in a more than doubled plasma LDL-C levels. In about 10% of FH patients, the cause is a mutation in the gene-encoding apolipoprotein B, which is the major protein of LDL particles. In less than 1%, a mutation in the gene-encoding proprotein convertase subtilisin/kexin type 9 (PCSK9) which is involved in degrading LDLR, is identified. In the rare autosomal recessive form (ARH), homozygous mutations in LDL receptor adaptor protein (LDLRAP1) gene can also result in an FH phenotype. The estimated prevalence of heterozygous FH (HeFH) is reported to be 1:200–500, but recent reports have showed an even higher prevalence such as 1:100–250 [2]. Such a prevalence would expect to yield over 4.5 million patients in Europe and 35 million patients worldwide, of whom 20–25% are children and adolescents [2, 3].

Despite an increasing knowledge of the clinical hallmarks of FH, elevated LDL-C levels, family and personal history of premature CHD, most cases of FH are not yet being recognized, and most remain under-treated [3].

### Phenotypic expression of FH in children and adolescents

In childhood, typical physical characteristics of FH, like corneal arcus, xanthelasma and xanthomas, especially in the Achilles tendons, in the extensor tendon on the dorsum of the hand and interdigital, are rare. If one or more are present, this results to be almost pathognomonic for FH. However, pain in the Achilles tendon is much more present [2, 4].

Although cardiovascular events are rare in childhood, children with FH show functional and morphological changes in the vessel wall. Carotid intima-media thickness (cIMT) and flow-mediated dilatation (FMD), both surrogate markers for atherosclerosis, are respectively increased and impaired in children with FH compared to healthy controls. The difference in mean cIMT between children with FH and unaffected siblings may even be significant as early as the age of 8 years, according to recent data [5].

In HoFH patients LDL-C levels exceed 500 mg/dL (13 mmol/L), xanthomas are evident also at the age of 2–5 years and the onset of cardiovascular disease (CVD) early in life can be disabling at a young age, also leading to a shortened life expectancy [2].

### Screening for HeFH in children

Children can be suspected of FH in at least three situations: a child from a family where HeFH has been identified or suspected (clinical/genetic criteria); a child from a family with a history of premature (before the age of 55 and 60 years in men and women, respectively) CVD, or a child with one of both parents being affected by primary hypercholesterolemia. This emphasizes the importance of assessing the family history regarding cholesterol levels, CVD and confirmed or suspected related conditions for all children [2, 6]. Screening strategies are summarized in Table 1 (Table1).

**Table 1:** Screening strategies for Familial Hypercholesterolemia (FH) in children and adolescents, adapted from Pederiva et al. [4]

● Universal screening: population screening for a specific age group.
● Selective screening: screening for a specific (high-risk) population.
● Cascade screening: from an index case (parent) to family members (including children).
● Reverse screening: from an index case (child/adolescent) to other family members.
● Child-parent screening: from children screened at a specific age to parents.

National screening for cholesterol values, soon at birth or later on, is currently not recommended. Although such universal screening is technically feasible in the context of neonatal screening on filter paper or later during the school years, it may be expensive (especially if the option of genetic analyses is chosen), raises a number of ethical issues, and needs governmental decision and support.

On the other hand, selective screening should be recommended after the age of 2 years. Firstly, plasma cholesterol levels are lower at birth and increase rapidly in the first weeks of life, and then gradually until 2 years of age, when lipid levels become quite constant up to the adolescent growth spurt, being almost similar to those seen in young adults. Secondly, literature studies do not recommend any dietary treatment up to the age of 2 years. And finally, the younger the children are, the less the chance of overlap between LDL-C levels in subjects with and without HeFH. Ideally, the screening should be proposed between 2 and 10 years of age. Indeed, dietary habits are more difficult to correct after this age, and a large variation in LDL-C (with average 10–20% fall) values occurs during adolescence, with increasing risk of false negative results.

In recent years, many authors have highlighted that cascade screening of close relatives is generally highly acceptable and does not impact on the quality of life. Cascade screening starts with drawing a pedigree, with the highlighting of subjects with a cardiovascular event or hypercholesterolemia (clinical or genetically defined) within the family tree. In the presence of FH, hypercholesterolemia or the cardiovascular event is recognizable in all generations, both in first and second degree relatives. The request for relatives in pharmaceutical therapy for hypercholesterolemia is also important [2, 6–9].

### Diagnosis of FH in the paediatric population

Diagnosis in children should preferably be established by the detection of FH-causing mutation, which is the gold standard for diagnosis. Genetic testing, however, is not always available. In that case FH in children can be diagnosed phenotypically by the presence of an increased LDL-C level plus a family history of premature CVD or elevated LDL-C levels compatible with FH [4, 10]. Recently, the European Atherosclerosis Society (EAS) proposed HeFH diagnostic criteria for children based on LDL-C cut-off values and family history of hypercholesterolemia and/or premature CVD [2]. The diagnostic algorithm of FH is summarized in Table 2 (Table 2).

**Table 2:** Diagnosis of FH in children and adolescents, adapted from Wiegman et al. [2]

● The most important key selective screening points are positive family history for premature CHD and elevated LDL-C levels
● Phenotypic diagnosis should be made using blood cholesterol testing.
LDL-C > 190 mg/dL on two different blood samples performed at baseline and after a three-months period of nutrition and lifestyle treatment is highly suggestive of a diagnosis of FH.
LDL-C > 160 mg/dL and a positive family history of premature CHD in first degree relative and/or high blood cholesterol in first degree relative indicates a highly probable diagnosis of FH.
LDL-C > 130 mg/dL and a parent with genetic diagnosis of FH is indicative of probable FH.
● Secondary causes of hypercholesterolaemia should be ruled out.
● DNA testing is the gold standard of the diagnosis. When a pathogenic LDL-R mutation is found in a first degree relative, children and/or adolescent should also be genetically tested.
● In case of a parent’s death for CHD, a child with hypercholesterolaemia (even if mild) should be tested genetically for FH and Lp(a) levels should be assayed.

It is increasingly recognized that childhood and early adolescence offer the most favorable timeframe for diagnosing FH, and also for introducing and maintaining lifelong treatment and management strategies. To achieve such radical care from a young age will require a shift in community and health professional perceptions of FH and its effects on the young. Little attention has been given to data for FH screening in general practice and by paediatricians, who should be able to detect the majority of affected subjects [11].

In countries with a history of dedicated screening programs, such as the Netherlands and Norway, the numbers in terms of newly diagnosed FH index cases and cascade tested relatives are much higher than in countries lacking any formal screening program. The Dutch cascade screening program, carried out between 1994 and 2014 using the services of genetic field workers, was very successful. In Slovenia the use of universal screening for children aged above 5 years has been introduced to help with the detection of FH, but the practicalities and cost-effectiveness remain to be better assessed. In the United States universal screening of cholesterol values at age 9–11 years has been endorsed by the American Academy of Paediatrics and the National Lipid Association (NLA), but has been incompletely undertaken and cost benefit analyses of this approach have not been performed [2].

Wald et al. examined the efficacy and feasibility of child-parent screening for FH in primary care practices, at routine immunization attendances by children aged 1–2 years in the UK over a three-years period. The child provided the screening entry point at an age identified as appropriate for the measurement of cholesterol. Once the child is identified as having FH, one of the parents will also harbour this condition, enabling two generations to be effectively screened as a part of the process. By screening children, most FH-positive adults subsequently identified should still be relatively young (average age of parent/sibling of parent is about 30 years), providing the opportunity for preventive treatment, prior to the onset of ischaemic heart disease events [9].

Cascade screening of close family relatives of known index cases is recognized as the most efficient and cost-effective approach for identifying new FH patients. The evidence to support cascade testing of relatives is based on specialist center approaches rather than on screening from primary care. The Dutch FH program which sought to find all FH patients, was centrally controlled and involved all specialists in cardiovascular care as well as general practitioners and had extensive media and scientific journal exposure to increase awareness in the general population and health professionals [12].

The improvement of public awareness about FH, especially in the community setting, needs to be addressed. Many families may be aware of premature CVD death in their own households, but the significance of these past events and the potential future risk for their own health is often not fully grasped. Young offsprings of affected patients are likely to feel entirely healthy and lacking in symptoms, and see no reason to commence life-long treatment for a condition they perceive as posing no immediate or potential threat [13]. In this context, all screening strategies to identify children with FH and initiate early lipid management are characterized by low adherence by the medical community and limited compliance by parents and children.

Despite increasing knowledge about the prevalence and risks of FH, many health professionals do not make any link between FH and the patient's clinical condition. A better appreciation of the underlying genetic nature of the disease and the fact that it will not be solely responsive to dietary and lifestyle intervention is needed. Health professionals caring for children and adolescents and paediatricians should always collect and document a family history of hypercholesterolemia or early CVD during control visits, specifically looking at high-risk children and families. Even though atherosclerosis has its origins in childhood, it is still considered an adult disease, predominantly because cardiovascular events do not usually occur for several decades, long after patients have left paediatric services [14].

**Fig. 1:**
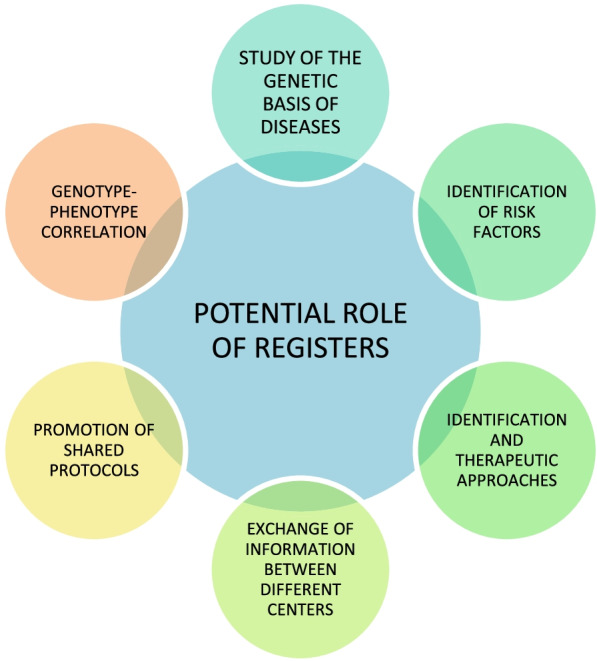
Potential role of pathology registers, adapted from Gazzotti et al. [15]

In recent years, FH Registers have been instituted in many countries, with the aim of generating large scale robust data on how FH is detected and managed. The potential of FH Registers and improved coding for FH needs to be linked to screening approaches, and definition and harmonization of the clinical diagnosis [15] (Fig. 1).

In Italy there is a specific network for the diagnosis and study of genetic dyslipidaemias. The LIPIGEN-FH (LIpid TransPort Disorders Italian GEnetic Network) study is an observational, multicentre, retrospective and prospective study established since 2009 to promote the diagnosis of genetic dyslipidaemias, with a primary focus on FH. In 2018 LIPIGEN Paediatric Gorup was founded, on the basis of the pre-existent LIPIGEN register. The main aim of this sub-study, including both paediatric and adult centers that (even occasionally) manage FH subjects under 18 years, is to improve detection, diagnosis, and management of children and adolescents affected by FH. The LIPIGEN Paediatric Group at present includes 31 Lipid Centers all over Italy [15].

### Expert commentary

Atherosclerosis may begin from conception, with evidence that in utero exposure to maternal high blood cholesterol impacts on arterial biology in the fetus [16]. Autopsy and imaging studies demonstrate that the atherosclerotic process starts in childhood and progresses in direct relation to plasma LDL-C levels [5]. This strengthens the need for early treatment of children with FH to reduce the impact on the cumulative life-burden of LDL-C. These concepts, which have been largely developed from epidemiologic evidence, have not always been simple to implement in the paediatric clinical practice. However, while individuals with FH who are treated from young age can have a normal life expectancy, FH remains largely under-diagnosed especially in the young population.

The reasons to screen for FH are simple and evident: it is such an extremely common metabolic disorder, affected patients die from this disease if untreated, and a highly effective treatment is available. The fact that FH is asymptomatic in children results in important adverse outcomes and, on the other side, very often paediatricians neglect to document the family history of hypercholesterolemia or early CVD, which is the cornerstone of selective screening. This emphasizes the importance that any physician, when visiting children (whatever the reason), assesses the family history with regard to cholesterol levels, CVD and confirmed or suspected genetic conditions [17].

An important issue, with respect to cholesterol screening, concerns the age at which screening might be most effective. LDL-C may decrease during puberty and then increase after puberty: thus, lipid values during puberty may be misleading with respect to the likelihood of FH.

Identifying children and adolescents with FH is important, but what is the best approach? Screening just prior to the onset of puberty or in early puberty may be the best choice, as those lipid levels may best reflect values in adulthood; nevertheless, puberty occurs at different age during adolescence. Furthermore, though periodical health checks are recommended throughout 5–11 years of age, many children in that age range do not comply to regular visits during which lipid screening might be performed [4].

Another issue to consider is that the atherosclerotic process begins to accelerate at around 10 years of age.

There are many factors indicating that cholesterol screening should be useful to identify FH children, but there are clearly logistical issues to consider. Hopefully, the discussion about cholesterol screening should address the identification of the important genetic entity FH, rather focusing on the individuals who are at highest lifetime risk of CVD, than on the role that obesity and other environmental factors may play. It is important to identify and develop clear approaches and treatment strategies which need to be pragmatic and context specific [18].

Optimal care for children and adolescents with FH requires a multidisciplinary framework including primary care professionals, paediatricians, and adult physicians.

The current screening strategy in Italy is not universal screening but target cascade screening for the identification of subject at high cardiovascular risk. In Italy, every child should have a family doctor, that is to say a dedicated paediatrician that follows the child from birth to adolescence not only for illnesses, but also for growth and neurodevelopmental monitoring and for non communicable disease prevention. In this context, the collection of a detailed and problem tailored family history for cardiovascular events in first and second degree relatives is a fundamental milestone for cardiovascular disease prevention, so as the detection of children at high cardiovascular risk and the prescription of a lipid profile for these children [19].

Patient support groups and networks have a critical role in improving FH children’s care. Empowering patients’ care may raise the awareness of FH in the community and improve collaboration between patient groups and the medical/scientific world.

Registers facilitate research and education and lead to better patients’ health outcomes.

The start of atherosclerosis prevention early in childhood is likely to result in a greater reduction in CVD morbidity and mortality than that achieved with current efforts aimed at preventing events in older adults [19].

## Conclusions

Early identification of children with FH is important to prevent atherosclerosis at the earliest stage of development, enables intervention at an early stage, when maximum benefit can still be obtained via lifestyle adaptations and therapy. Early detection and treatment of FH in childhood and adolescence will gain decades of healthy life.

During the last 20 years, a lot of experience has been gained, and the means to solve most issues encountered in screening programs have been published; however, several evidence gaps need to be filled. This can improve existing models of screening and care for children with FH, which need to be context specific.

A multidisciplinary framework integrating primary care, pediatricians and adult physicians aiming at optimal models of detection and care will ultimately change the natural history of this common and life-threatening disease [2].

## Data Availability

Not applicable.

## Abbreviations

ARH: Autosomal Recessive Hypercholesterolaemia
CHD: Coronary Heart Disease
cIMT: Carotid intima-media thickness
CVD: Cardio Vascular Disease
FH: Familial Hypercholesterolaemia
FMD: Flow Mediated Dilation
HDL: High Density Lipoprotein
HeFH: Heterozygous Familial Hypercholesterolaemia
HoFH: Homozygous Familial Hypercholesterolaemia
LDL: Low Density Lipoprotein
LDLRAP1: LDL receptor adaptor protein
NLA: National Lipid Association
PCSK9: Proprotein convertase subtilisin/kexin type 9

## References

[1] Fahed AC, Nemer GM (2011). Familial hypercholesterolemia: the lipids or the genes?. Nutr Metab.

[2] Wiegman A, Gidding SS, Watts GF, Chapman MJ, Ginsberg HN, Cuchel M (2015). Familial hypercholesterolaemia in children and adolescents: gaining decades of life by optimizing detection and treatment. Eur Heart J.

[3] Nordestgaard BG, Chapman MJ, Humphries SE, Ginsberg HN, Masana L, Descamps OS (2013). Familial hypercholesterolaemia is underdiagnosed and undertreated in the general population: guidance for clinicians to prevent coronary heart disease: consensus statement of the European Atherosclerosis Society. Eur Heart J.

[4] Pederiva C, Capra ME, Viggiano C, Rovelli V, Banderali G, Biasucci G (2021). Early prevention of atherosclerosis: detection and management of Hypercholesterolaemia in Children and Adolescents. Life.

[5] Kusters DM, Wiegman A, Kastelein JJ, Hutten BA (2014). Carotid Intima-Media thickness in children with familial Hypercholesterolemia. Circ Res.

[6] Goldberg AC, Hopkins PN, Toth PP, Ballantyne CM, Rader DJ, Robinson JG (2011). Familial hypercholesterolemia: screening, diagnosis and management of pediatric and adult patients: clinical guidance from the National Lipid Association Expert Panel on Familial Hypercholesterolemia. J Clin Lipidol.

[7] Pyles LA, Elliott E, Neal WA (2017). Screening for Hypercholesterolemia in children: what strategies can be employed. Curr Cardiovasc Risk.

[8] Newson AJ, Humphries SE (2005). Cascade testing in familial hypercholesterolaemia: how should family members be contacted?. Eur J Hum Genet.

[9] Wald DS, Bestwick JP, Wald NJ (2007). Child-parent screening for familial hypercholesterolaemia: screening strategy based on a meta-analysis. BMJ (Clinical research ed).

[10] Expert Panel On Integrated Guidelines for Cardiovascular Health and Risk Reduction in Children and Adolescents. Expert Panel on Integrated Guidelines for Cardiovascular Health and Risk Reduction in Children and Adolescents: Summary Report. Pediatrics. 2011;128:S213–S256. 10.1542/peds.2009-2107C. 10.1542/peds.2009-2107C PMC4536582 22084329

[11] Brett T, Qureshi N, Gidding SS, Watts GF (2018). Screening for familial hypercholesterolaemia in primary care: time for general practice to play its part. Atherosclerosis.

[12] Kerr M, Pears R, Miedzybrodzka Z, Haralambos K, Cather M, Watson M (2017). Cost effectiveness of cascade testing for familial hypercholesterolaemia, based on data from familial hypercholesterolaemia services in the UK. Eur Heart J.

[13] Andersen LK, Jensen HK, Juul S, Faergeman O (1997). Patients' attitudes toward detection of heterozygous familial hypercholesterolemia. Arch Intern Med.

[14] Sorubarajan T, Lewis BD, Burnett JR, Martin AC (2017). Documenting family history in children with hypercholesterolaemia: a lost opportunity. J Paediatr Child H.

[15] Gazzotti M, Casula M, Olmastroni E, Averna M, Arca M, Catapano AL (2020). How registers could enhance knowledge and characterization of genetic dyslipidaemias: The experience of the LIPIGEN in Italy and of other networks for familial hypercholesterolemia. Atherosclerosis Suppl.

[16] Napoli C, Christopher KG, Witztum JL, Deutsch R (1999). Influence of maternal hypercholesterolaemia during pregnancy on progression of early atherosclerotic lesions in childhood: Fate of Early Lesions in Children (FELIC) study. Lancet.

[17] Futema M, Cooper JA, Charakida M, Boustred C, Sattar N, Deanfield J (2017). Screening for familial hypercholesterolaemia in childhood: Avon Longitudinal Study of Parents and Children (ALSPAC). Atherosclerosis.

[18] Catapano AL, Graham I, De Backer G, Wiklund O, Chapman MJ, Drexel H (2016). 2016 ESC/EAS guidelines for the management of dyslipidaemias the task force for the management of dyslipidaemias of the European Society of Cardiology (ESC) and European Atherosclerosis Society (EAS) Developed with the special contribution of the European Association for Cardiovascular Prevention & Rehabilitation (EACPR). Atherosclerosis.

[19] Capra ME, Pederiva C, Banderali G, Biasucci G (2021). Prevention starts from the crib: the paediatric point of view on detection of families at high cardiovascular risk. Ital J Pediatr.

